# Special Issue “Integrated Approaches, Molecular Mechanisms and Therapies in Ocular Surface Diseases”

**DOI:** 10.3390/ijms27115106

**Published:** 2026-06-04

**Authors:** Marco Zeppieri, Mario Damiano Toro, Caterina Gagliano

**Affiliations:** 1 Department of Ophthalmology, University Hospital of Udine, 33100 Udine, Italy; 2 Department of Medicine, Surgery and Health Sciences, University of Trieste, 34127 Trieste, Italy; 3 Eye Clinic, Public Health Department, Federico II University, Via Pansini 5, 80131 Naples, Italy; mariodamiano.toro@unina.it; 4 Department of Special Surgery, University of Jordan, Queen Rania St., Amman 11942, Jordan; 5 Department of Medicine and Surgery, University of Enna “Kore”, Piazza dell’Università, 94100 Enna, Italy; caterina.gagliano@unikore.it; 6 Mediterranean Foundation “G.B. Morgagni”, 95125 Catania, Italy

The ocular surface is a highly specialized and dynamic complex system, characterized by close interconnections among epithelial integrity, immunological privilege, and tear film homeostasis, all of which are essential for proper visual function. Any alteration of this balance, whether caused by environmental factors, inflammatory and/or immune-mediated processes, or genetic factors, may lead to a wide range of anterior ocular surface diseases, which remain a major cause of morbidity worldwide. In recent years, there has been growing interest in integrated approaches that combine molecular discoveries with clinical and translational strategies, with the aim of better understanding the underlying pathogenetic mechanisms and offering increasingly targeted therapies, rather than treatment directed only toward symptomatic management. In light of this, this Special Issue aims, first, to analyze emerging molecular pathways and, second, to explore new and potential therapeutic interventions and clinically relevant applications of regenerative and biologically inspired treatments. Particular attention will be given to blood-derived and blood-component-derived interventions and therapies, and to their increasingly relevant and recognized roles in ocular surface reconstruction and health.

The articles selected for this Special Issue illustrate the multifactorial nature of ocular surface disease, encompassing experimental models, translational research, clinical innovation, and genetic characterization. Taken together, these contributions outline a coherent narrative connecting disease biology with therapeutic applications. From this body of evidence, a central theme emerges: the effective management of ocular surface diseases requires, on the one hand, modulation and treatment of inflammation and, on the other, restoration of epithelial homeostasis, regulation and inhibition of pathological angiogenesis, and promotion of re-epithelialization and tissue regeneration. This integrated approach is particularly evident in experimental models of corneal injury, where the intersection of inflammatory cascades, tissue and cellular stress responses, and anatomical and structural remodeling processes is clearly demonstrable and clinically relevant.

Hu et al. conducted a thorough analysis of the adhesion mechanisms, biomedical uses, and existing limitations of ocular tissue adhesives in corneal healing and ocular surface reconstruction. The authors examined both physical and chemical adhesion techniques, emphasizing the growing significance of tissue adhesives in wound closure, drug administration, regenerative scaffolds, and biologically inspired eye therapies. Volatier et al. examined the function of ATG7-mediated autophagy in maintaining corneal epithelial homeostasis through a conditional knockout animal model. Their findings indicated that compromised autophagy correlated with epithelial thickening, modified lymphatic responses, and disruption of corneal immune privilege, reinforcing the notion that intracellular stress-response pathways significantly influence ocular surface integrity and inflammatory regulation.

Zeppieri et al. assessed the therapeutic effectiveness of allogeneic umbilical cord blood serum eye drops in patients with severe dry eye disease unresponsive to standard treatment. The research demonstrated significant improvements in both subjective symptoms and objective ocular surface metrics across multiple severe disease subtypes, reinforcing the growing translational interest in blood-derived regenerative treatments for complex ocular surface conditions. Charoenrook et al. provided a comprehensive clinical and molecular analysis of two families afflicted by Meesmann epithelial corneal dystrophy, linked to both established and novel mutations in KRT3 and KRT12. The study enhanced existing genotype–phenotype correlations through multimodal imaging and targeted next-generation sequencing, highlighting the diagnostic significance of combining sophisticated imaging with molecular investigation in inherited corneal diseases. Bhujel et al. investigated the therapeutic effects of rapamycin in an experimental mouse model of corneal alkali burn, comparing its efficacy to that of cyclosporine A. The study indicated that rapamycin diminished inflammatory signaling, neovascularization, fibrosis, and epithelial disruption, while enhancing tear film stability and corneal healing, suggesting a broader modulatory influence on ocular surface repair processes following severe injury.

Among the experimental studies we analyzed, interesting insights into pathological mechanisms and therapeutic approaches come from the analysis of rapamycin in murine models of corneal alkali burns. The authors showed that, through the regulation of key inflammatory pathways, particularly by inhibiting NF-κB signaling, rapamycin exerts important protective effects, reducing the production of pro-inflammatory cytokines, neovascularization, and inflammatory/immune cell infiltration. Moreover, through multifactorial mechanisms of action beyond the immunosuppressive activity described above, rapamycin appears to play a key role in maintaining epithelial barrier integrity, preserving differentiation markers, and supporting tear film function. Overall, these findings show that, rather than acting on individual components of the inflammatory response, it is plausible and desirable to target upstream biological regulators of inflammatory pathways that can produce multiple downstream effects.

In order to establish a fundamental and essential link between anterior ocular surface tissue disease and intracellular homeostasis, it is of primary importance to analyze and identify, at both the cellular and biomolecular levels, the mechanisms of autophagy in the corneal epithelium. Studies performed in ATG7-deficient mice have shown that impairment of the autophagic process leads to irreversible structural changes in the corneal epithelium, increased lymphangiogenesis, and the creation of a pro-inflammatory environment, with impairment of the immunological privilege normally present at the corneal level. Autophagy, therefore, appears to play a crucial role in modulating the inflammatory response and preserving epithelial function and integrity; above all, it may represent an underestimated contributing factor in the development of ocular surface disease. Furthermore, the identification of compensatory proteostatic mechanisms demonstrates the complexity of cellular adaptive capacity and suggests that these systems may potentially be exploited for new targeted therapeutic opportunities.

Studies analyzing regenerative therapies, particularly the use of blood and blood derivatives, further reinforce the clinical importance of these mechanistic observations. One study evaluated the use of allogeneic umbilical cord blood serum eye drops, providing concrete and convincing evidence of their efficacy in reducing inflammation, promoting re-epithelialization, and improving both objective and subjective outcomes in severe forms of dry eye disease. Umbilical cord blood-based eye drops contain a complex mixture of cytokines, growth factors, and other beneficial substances, and therefore represent a plausible and rational biological therapy to improve tear film stability and treat defects in epithelial healing. Indeed, the study shows that this approach is not only useful and effective in refractory and complex cases but also highlights its broader and translational potential as a regenerative therapy in ocular surface medicine. However, significant obstacles to wider clinical application and implementation remain: the main issues that need to be resolved concern patient selection, standardization of preparation techniques, and medium- to long-term safety.

Scientific advances in imaging and genetic technologies are simultaneously transforming the key conceptual and diagnostic paradigms of ocular surface diseases. An important clinical–genetic study included in this Special Issue on Meesmann corneal dystrophy highlights the need to integrate next-generation sequencing techniques with new high-resolution diagnostic imaging modalities. This is essential for a better understanding, and therefore correlation, of genotype and phenotype. The discovery of new mutations and, consequently, their correlation with complex epithelial corneal phenotypes highlights the complexity and heterogeneity of the spectrum of inherited corneal dystrophies and underscores the growing need for precise diagnosis. Therefore, both in vivo corneal confocal microscopy and anterior segment optical coherence tomography have become useful tools for assessing the corneal epithelium in a detailed and effective manner and for obtaining information that supports accurate diagnosis and, consequently, appropriate therapeutic management.

An extensive literature review on various ocular tissue adhesives, included in this Special Issue, broadens this field by analyzing innovative new biomaterials designed to repair corneal damage and improve healing. These new adhesive biomaterials do not simply seal the wound, but have multiple functions, including antimicrobial activity, scaffold support to promote tissue repair, and drug delivery. These new bioengineered solutions represent a radical paradigm shift made possible by the development of new materials: physiological processes are “imitated,” while medicine moves toward an increasingly harmonious integration with the patient’s own tissues. It is therefore reasonable to conclude that these approaches may represent a potentially significant new treatment for corneal lesions, post-surgical sequelae, and other conditions affecting the anterior segment, particularly in complex and refractory cases that are difficult to manage with conventional therapies.

However, numerous challenges remain, despite the many advances addressed in this Special Issue. The translation of experimental findings into everyday clinical practice is not straightforward: the process is often hindered by the fact that studies are conducted in heterogeneous populations, by the lack of standardized outcomes and measures, and by substantial differences among disease models. Other important issues include regulatory approval, economic sustainability, and scalability, although biologically derived products and regenerative therapies show great potential. Long-term studies will therefore be needed to assess the true duration of therapeutic effects and to identify adverse events in the medium and long term. With clear, shared clinical standards and advances in technology, it will likely become possible to apply molecular diagnostics in routine clinical practice. The main interactions among molecular pathways, tissue alterations, and innovative therapies are summarized in Figure 1, underscoring the need for a multidisciplinary and interdisciplinary approach in the management of ocular surface diseases.

In the future, the integration of precision medicine, regenerative medicine, and molecular biology is likely to become central to the treatment of ocular surface diseases [1]. Advances in transcriptomics and proteomics, and more generally in omics technologies, may improve our understanding of the pathophysiological mechanisms underlying disease and lead to the identification of new therapeutic targets [2]. This may enable the development of increasingly personalized therapies, guided by patients’ genetic and molecular characteristics, leading to more effective interventions [3]. To achieve this, it is clear that collaboration between ophthalmologists and specialists in immunology and biotechnology will be essential to drive innovation forward and translate scientific discoveries into clinically applicable therapies with tangible benefits [4,5].

This Special Issue aims to provide the broadest possible overview of recent advances in research, emphasizing the importance of linking biomolecular mechanisms to clinical practice. As highlighted by the studies presented, effective management of ocular surface diseases requires an approach that focuses on treating inflammation, restoring epithelial integrity, promoting tissue regeneration, and modulating the immune system. By integrating diverse perspectives and methodologies, this Special Issue seeks to deepen understanding of the biological and biomolecular mechanisms of the ocular surface and lay the groundwork for future diagnostic and therapeutic strategies.

## Figures and Tables

**Figure 1:**
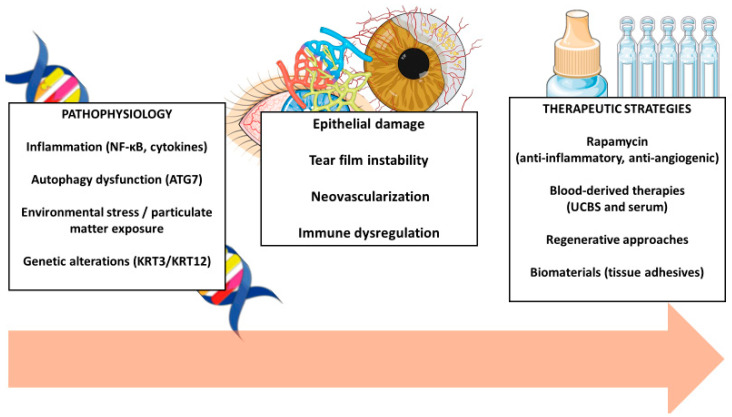
Flowchart: From Ocular Surface Pathology to Treatment. The figure briefly summarizes the pathophysiology of ocular surface disease, highlighting the complex interactions among genetic, environmental, and biological factors and how these factors collectively alter cells and tissues. These mechanisms underlie the pathological processes of angiogenesis, immune dysregulation, and tear film instability. Finally, potential new therapies are highlighted, including rapamycin (anti-inflammatory and anti-angiogenic), new biomaterials, and blood derivatives. Image(s) provided by Servier Medical Art (https://smart.servier.com), licensed under CC BY 4.0 (https://creativecommons.org/licenses/by/4.0/).
